# Inhibition of red blood cell development by arsenic-induced disruption of GATA-1

**DOI:** 10.1038/s41598-020-76118-x

**Published:** 2020-11-04

**Authors:** Xixi Zhou, Sebastian Medina, Alicia M. Bolt, Haikun Zhang, Guanghua Wan, Huan Xu, Fredine T. Lauer, Shu Chun Wang, Scott W. Burchiel, Ke Jian Liu

**Affiliations:** 1 Department of Pharmaceutical Sciences, The University of New Mexico College of Pharmacy, Albuquerque, NM 87131 USA; 2 Department of Biology, New Mexico Highlands University, Las Vegas, NM 87701 USA; 3 School of Pharmacy, East China University of Science and Technology, Shanghai, 200237 China; 4 Institute of Hematology and Blood Diseases Hospital, Chinese Academy of Medical Sciences and Peking Union Medical College, Tianjin, 300020 China

**Keywords:** Metals, Mechanisms of disease

## Abstract

Anemia is a hematological disorder that adversely affects the health of millions of people worldwide. Although many variables influence the development and exacerbation of anemia, one major contributing factor is the impairment of erythropoiesis. Normal erythropoiesis is highly regulated by the zinc finger transcription factor GATA-1. Disruption of the zinc finger motifs in GATA-1, such as produced by germline mutations, compromises the function of this critical transcription factor and causes dyserythropoietic anemia. Herein, we utilize a combination of in vitro and in vivo studies to provide evidence that arsenic, a widespread environmental toxicant, inhibits erythropoiesis likely through replacing zinc within the zinc fingers of the critical transcription factor GATA-1. We found that arsenic interacts with the N- and C-terminal zinc finger motifs of GATA-1, causing zinc loss and inhibition of DNA and protein binding activities, leading to dyserythropoiesis and an imbalance of hematopoietic differentiation. For the first time, we show that exposures to a prevalent environmental contaminant compromises the function of a key regulatory factor in erythropoiesis, producing effects functionally similar to inherited GATA-1 mutations. These findings highlight a novel molecular mechanism by which arsenic exposure may cause anemia and provide critical insights into potential prevention and intervention for arsenic-related anemias.

## Introduction

Anemia is a hematological disorder that affects over 25% of the global population^1–3^. A major cause of anemia is decreased red blood cell production^4–6^, which occurs primarily in the bone marrow. Red blood cells are produced through erythropoiesis starting from hematopoietic stem cells (HSC)^7,8^. The lineage commitment and differentiation of HSC is a sophisticated process regulated by a series of factors. HSC differentiate to common myeloid progenitors (CMP), which depending on expression levels and subsequent functional antagonism of two key transcriptional regulators, GATA-1 and PU.1, commit to either erythro-megakaryocytic or myeloid lineages, respectively^9–13^.

Once committed to the erythro-megakaryocytic lineage, megakaryocyte-erythroid progenitors (MEP) differentiate to megakaryocyte and erythroid progenitors^8^. The earliest committed erythroid progenitors are detected as slowly proliferating burst-forming unit erythroid cells (BFU-E). BFU-E differentiate into rapidly dividing colony-forming unit erythroid cells (CFU-E)^8^. CFU-E undergo four additional stages of differentiation to proerythroblast, basophilic erythroblast, polychromatophilic erythroblast, and orthochromatophilic erythroblast to finally produce mature red blood cells^8^.

Erythropoiesis is a dynamic and precisely regulated process under the control of many regulatory factors^14–16^, among which GATA-1 is recognized as the master regulatory factor and its activity is necessary for the normal differentiation of early erythroid progenitors (i.e. BFU-E, CFU-E, and proerythroblasts)^15,17,18^. GATA-1 is the founding member of the GATA family of transcription factors and is responsible for inducing and repressing many genes required for erythroid differentiation^14,18–21^. The function of GATA-1 depends on two zinc finger (ZF) domains^22^. The C- and N-terminal ZFs are responsible for binding to T/A(GATA)A/G DNA consensus DNA sequences and to an important co-factor, friend of GATA-1 (FOG-1), respectively^22–24^. Mutation, protein level alteration, or dysfunction of GATA-1 results in the inhibition of erythropoiesis^14,21,25–27^. Zinc finger dysfunction caused by mutations in the ZF motifs of GATA-1 is a characteristic phenotype of inherited dyserythropoietic anemia^25,26^. Similarly, myelopoiesis is regulated primarily by the actions of another key transcription factor, PU.1. Unlike GATA-1, PU.1 is not a zinc finger protein and is of the E Twenty-Six (ETS) family of transcription factors^28–30^. PU.1 binds to purine-rich DNA consensus sequences (GAGGAA), and is responsible for activating the expression of genes required for myelopoiesis and cellular communications of the immune system^12,13,30^.

Arsenic is a widespread environmental toxicant that poses a significant threat to public health. Millions of people worldwide are chronically exposed to inorganic trivalent arsenic (arsenite, AsIII) in drinking water at levels exceeding the World Health Organization and United States Environmental Protection Agency maximum contaminant level of 10 µg/L (ppb)^31,32^. Arsenic levels up to 1700 ppb have been measured in surface water in the United States^33^ and 12% of water supplies in the north central and western regions have levels > 20 ppb, with much higher levels occurring in unregulated well water^33,34^. Exposure to arsenic is well documented to produce many acute and chronic health effects, including anemia^32,35–40^. We recently reported that AsIII exposure correlates with a decrease in red blood cell counts in a group of men from rural Bangladesh exposed to a wide range of AsIII in their drinking water^38^. Our previous work also showed that low-level AsIII exposure causes anemia through the inhibition of erythropoiesis in mice^41^, but the molecular mechanisms of arsenic-caused anemia are still largely unknown.

Our work on AsIII carcinogenesis demonstrated that ZF DNA repair proteins, such as poly (ADP-ribose) polymerase 1 (PARP-1), are highly sensitive molecular targets of AsIII^42–45^. In these studies, we discovered that AsIII interacted with the ZF domain, causing zinc loss and dysfunction of the PARP-1 protein^42,43^. Other groups have reported similar findings of AsIII interaction with zinc finger proteins^46–48^. Zhang et al. found that arsenic binds directly to the cysteine residues in the zinc finger domains of promyelocytic leukemia protein causing conformational changes that enhance degradation of the protein^48^. It is important to note that not all zinc finger proteins are sensitive targets of arsenic. A key finding from our work was that AsIII selectively interacts with ZFs containing ≥ 3 cysteine residues^45^, making ZF proteins containing at least three cysteines, which are minority in ZF protein family, highly sensitive to AsIII exposure.

Inspired by our previous work as well as that of others documenting arsenic interactions with ZF proteins, we hypothesized that arsenic-induced inhibition of erythropoiesis may be attributed to loss of GATA-1 function, resulting from selective interaction of AsIII with the ZFs on GATA-1, which are C4 (4 cysteines) configuration. Herein, we report that GATA-1 is a sensitive molecular target of AsIII exposure at environmentally relevant concentrations. Our findings demonstrate, for the first time, that exposure to a widespread environmental toxic metal disrupts the function of GATA-1, the key regulator of red blood cell development, resulting in the inhibition of erythropoiesis. The results presented here highlight a novel mechanism by which AsIII exposure causes anemia, and also suggest that arsenic influences the lineage commitment of hematopoietic progenitor cells (HPC) by differential interactions with key regulatory proteins.

## Results

### AsIII inhibits erythropoiesis in vivo

**Figure 1:**
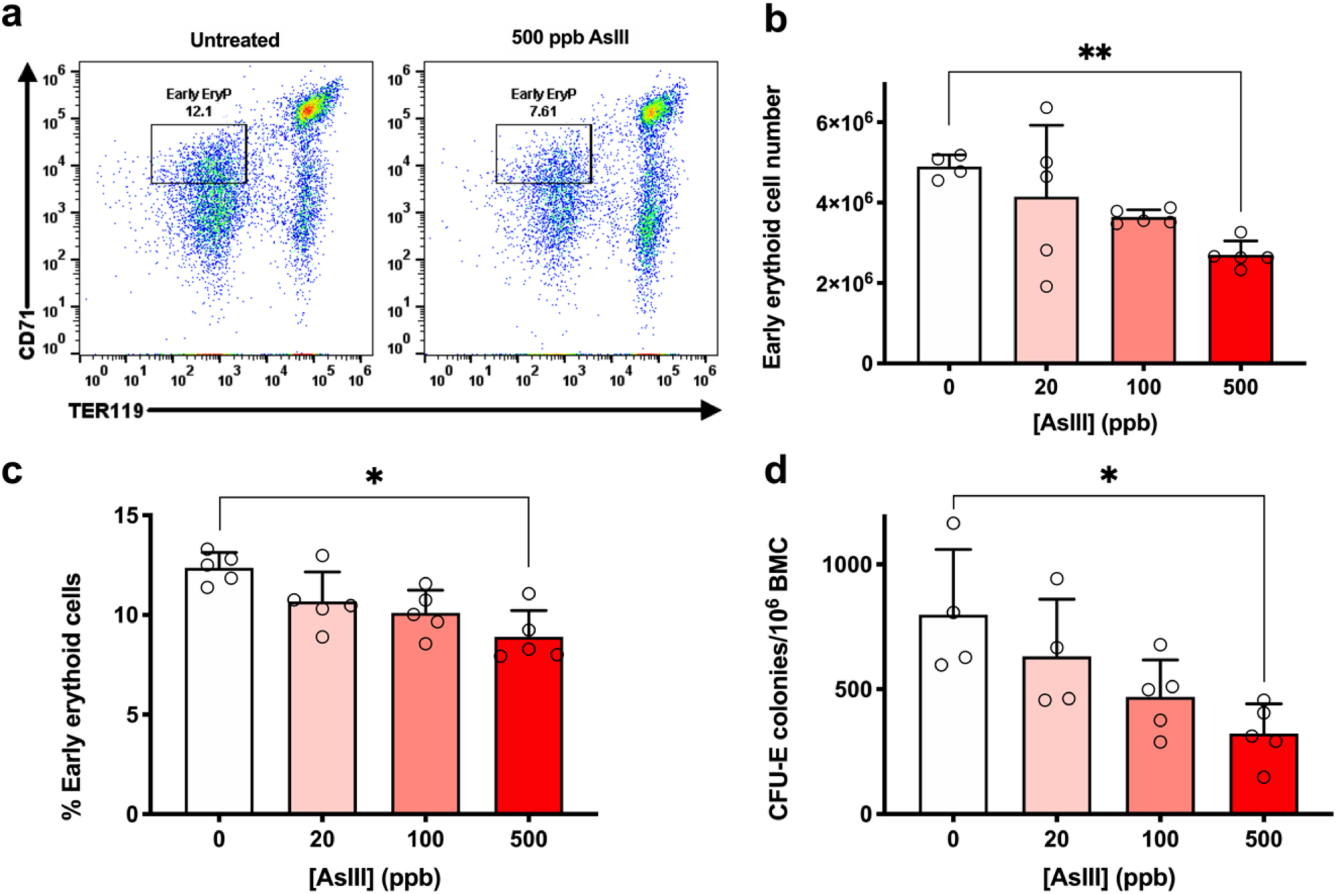
Inhibition of bone marrow erythropoiesis in vivo by AsIII exposure. Male C57BL/6 J mice were exposed to 0, 20, 100, and 500 ppb AsIII (0, 0.3, 1.3, and 6.7 µM, respectively) in drinking water for 30 days. Bone marrow cells were isolated from the femurs of each mouse and utilized for flow cytometry and CFU-E colony forming assays. (**a**) Representative flow cytometry dot plot depicting effects of 0 and 500 ppb AsIII on early erythroid progenitor cells (EryP; CD71^+^, Ter119^−^; late BFU-E and CFU-E) defined by CD71 and TER119 surface marker expression. (**b**) Total numbers or (**c**) percentages of early erythroid progenitor cells measured by CD71 and TER119 surface marker expression using flow cytometry. (**d**) Inhibition of CFU-E colony formation (shown as number of colonies/10^6^ bone marrow cells). Data are expressed as mean ± SD, *n* = 5 mice/group, *p < 0.05, ** *p* < 0.001 in one-way ANOVA, followed by Tukey’s post hoc test compared to no treatment group.

To demonstrate that AsIII exposure inhibits erythropoiesis in vivo, we exposed male C57BL/6J mice to 20, 100, and 500 ppb AsIII (0.3, 1.3, and 6.7 µM, respectively) via drinking water for 30 days. Early erythroid cells (CD71^+^/Ter119^−^; late BFU-E and CFU-E) were assessed in bone marrow by CD71 and Ter119 surface marker expression using flow cytometry^49,50^. AsIII exposure reduced the total number and percentage of early erythroid cells in a dose-dependent manner, starting from 0.02 µM (Fig. 1a–c). To further demonstrate that AsIII compromises the differentiation of early erythroid progenitors, we assessed the colony-forming ability of CFU-E from bone marrow of AsIII exposed mice. CFU-E colony formation was reduced by AsIII, starting at 20 ppb and was suppressed by over 60% with 500 ppb AsIII (Fig. 1d). These results provide evidence that in vivo drinking water exposure to environmentally relevant levels of AsIII suppress the differentiation of early erythroid progenitors in the bone marrow.

### AsIII inhibits erythropoiesis, but not myelopoiesis

**Figure 2:**
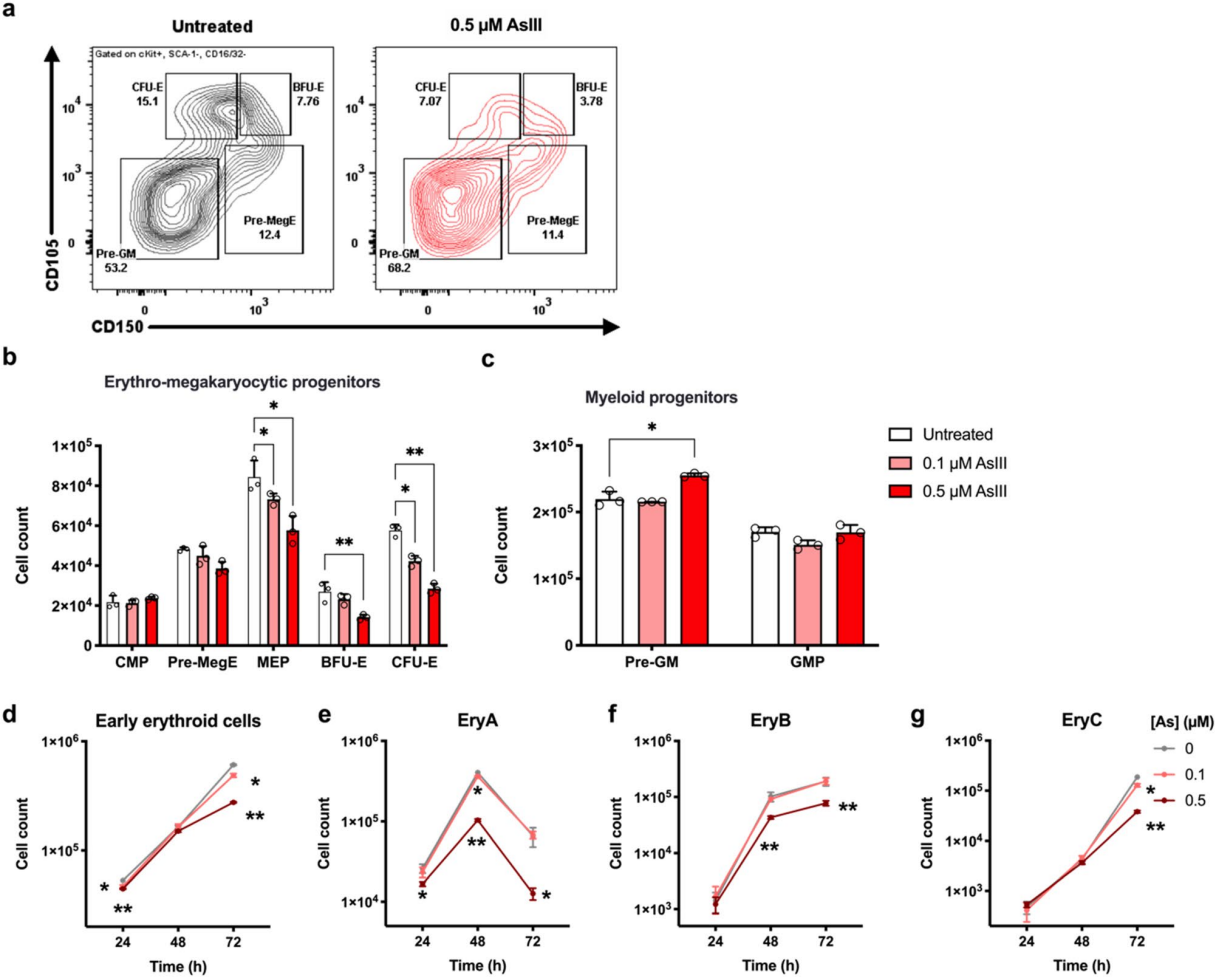
AsIII suppresses erythropoiesis, not myelopoiesis, of primary mouse bone marrow hematopoietic progenitor cells (HPC). Primary mouse bone marrow HPC were stimulated with erythropoietin and stem cell factor to induce erythroid differentiation in the presence of 0, 0.1, or 0.5 AsIII for 24 h. (**a**) Representative flow cytometry plot depicting effects of 0 and 0.5 μM AsIII on erythro-megakaryocytic and myeloid progenitor cell subsets. (**b**) Total numbers of surface marker defined erythro-megakaryocytic progenitors: CMP (Lin^−^, cKit^+^, SCA-1^−^, CD16/32^−^, CD34^+^); PreMegE (Lin^−^, cKit^+^, SCA-1^−^, CD16/32^−^, CD150^+^, CD105^−, low^); MEP (Lin^-^, cKit^+^, SCA-1^−^, CD16/32^−^, CD34^−^), BFU-E (Lin^−^, cKit^+^, SCA-1^−^, CD16/32^−^, CD150^+^, CD105^+^), CFU-E (Lin^−^, cKit^+^, SCA-1^−^, CD16/32^−^, CD150^−^, CD105^+^), and (**c**) myeloid progenitors: Pre-GM (Lin^−^, cKit^+^, SCA-1^−^, CD16/32^−^, CD150^−^, CD105^−^); GMP (Lin^−^, cKit^+^, SCA-1^−^, CD16/32^+^, CD150^-^) after 24 h exposure to AsIII. Total numbers of later-stage erythroblast subsets after 24, 48, and 72 h exposure to 0, 0.1, or 0.5 μM AsIII (**d**) early erythroblasts, CD71^low/high^, Ter119^−,low^, (**e**) basophilic (EryA), CD71^high^Ter119^high^FSC^high^, (**f**) late basophilic and polychromatic (EryB), CD71^high^Ter119^high^FSC^low^, (**g**) orthochromatic (EryC), CD71^low^Ter119^high^FSC^low^). Data are expressed as mean ± SD, *n* = 3, *p < 0.05, ** *p* < 0.001 in one-way ANOVA, followed by Tukey’s post hoc test compared to no treatment group.

As an effort to characterize the inhibitory effects of AsIII on erythropoiesis and to investigate whether AsIII impairs other hematopoietic lineages, we developed an in vitro model of erythropoiesis using primary mouse bone marrow HPC stimulated with erythropoietin (EPO) and stem cell factor (SCF)^51^. The progression of HPC through the stages of hematopoiesis were assessed based on surface marker phenotype using flow cytometry^52,53^. We identified a significant reduction in the numbers of erythro-megakaryocytic progenitors (MEP, *p* = 0.0114; BFU-E, *p* = 0.00063; and CFU-E, *p* = 0.0056) after 24 h exposure to 0.5 μM AsIII (Fig. 2a,b and Supplementary Fig. S1), but no significant changes in CMPs were observed with either AsIII dose (Fig. 2b). Additionally, the number of early myeloid progenitors (i.e. pre-granulocyte macrophage (pre-GM)) was significantly increased (*p* = 0.0192) with AsIII exposure (Fig. 2c and Supplementary Fig. S1).

These results show that the AsIII-induced inhibition of erythropoiesis starts from very early stages of erythroid differentiation. AsIII likely disrupts the transition of CMP to MEP and subsequent differentiation of early stages of erythroid progenitors (i.e. processes dependent on GATA-1), rather than reducing the CMP population directly. Interestingly, the suppression of erythropoiesis skews hematopoietic differentiation in favor of myelopoiesis, despite EPO stimulation and the lack of myeloid supportive growth factors.

To determine whether the suppressive effects of AsIII on early erythroid progenitor differentiation persist to later-stages of maturation, we evaluated the progression of erythroblast differentiation every 24 h for 72 h based on CD71 and Ter119 surface marker expression and cell size^49,50^. A significant suppression of early erythroblast subsets (late BFU-E, CFU-E, and proerythroblasts, *p* = 0.0042) and basophilic erythroblasts (EryA, *p* = 0.0162) was observed after 24 h exposure to 0.5 μM AsIII (Fig. 2d,e and Supplementary Fig. S1). These effects were persistent up to 72 h, with substantially fewer AsIII exposed erythroblasts properly transitioning to late stages of erythroblast maturation (late basophilic and polychromatic (EryB) and orthochromatic (EryC)) (Fig. 2f,g and Supplementary Fig. S1).

Collectively, these results suggest that AsIII inhibits early stages of erythropoiesis and these effects are persistent throughout erythroblast differentiation, resulting in decreased production of mature erythroblast subsets. However, the suppressive effects of AsIII were selective to erythro-megakaryocytic progenitors, as myeloid progenitors were not reduced by AsIII exposure.

**Figure 3:**
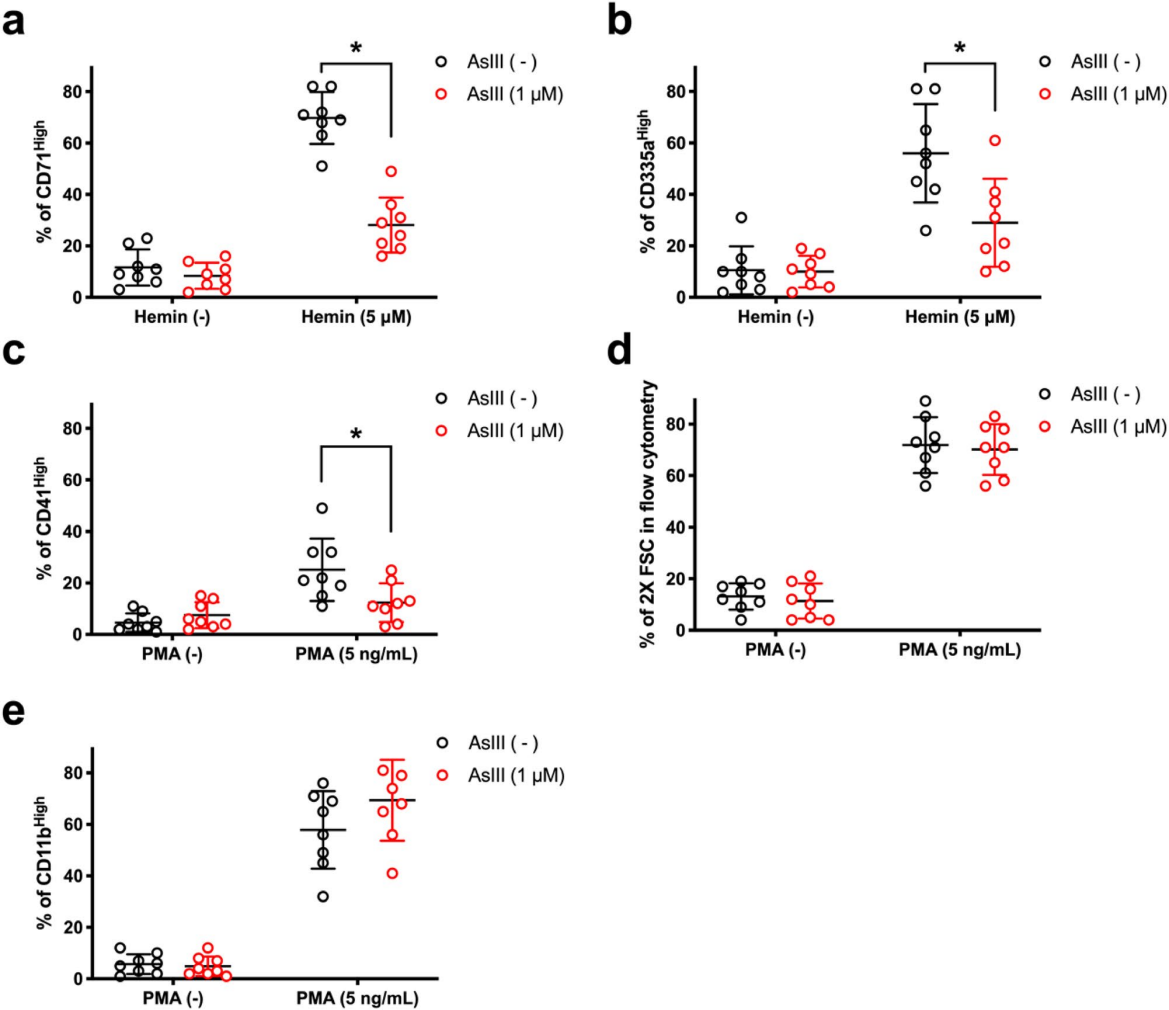
AsIII disrupts erythroid and megakaryocytic differentiation, not monocyte or myeloid differentiation of K562 cells. Human K562 erythroleukemia cells were treated with 1 μM AsIII for 48 h. Erythroid, megakaryocyte, monocyte, and myeloid differentiation was induced with hemin or PMA for 24 h, respectively. Percentages of erythroid differentiated K562 cells as indicated by (**a**) CD71^+^ or (**b**) CD235a^+^. (**c**) PMA-induced megakaryocyte differentiation of K562 cells (CD41^high^). (**d**) PMA-induced monocyte (CD11b^+^ and 2X forward scatter) and (**e**) myeloid differentiation CD11b^high^. Data are expressed as mean ± SD, *n* = 3, *p < 0.05 in two-tailed Student’s *t*-test compared to no treatment group.

To further demonstrate that AsIII selectively inhibits erythropoiesis, we tested the effects of AsIII on the hemin or PMA-induced erythroid^54^, megakaryocytic, myelocytic, and monocytic differentiation, respectively^54–57^. In hemin stimulated K562 cells, erythropoietic differentiation was measured using surface marker expression of CD71 and CD235a by flow cytometry. Since the K562 cell line has some resistance to arsenic^58^, slightly higher concentrations of AsIII were used in these experiments. AsIII at 1 µM significantly reduced the percentage of CD71^+^ and CD235a^+^ erythroid differentiated K562 cells (*p* = 0.0221 and 0.0469, respectively; Fig. 3a,b). Megakaryocytic differentiation of K562 cells was determined using CD41 surface marker expression by flow cytometry after exposure to 1 μM AsIII. AsIII significantly reduced the percentage of PMA differentiated megakaryocytic (CD41^high^) K562 cells (*p* = 0.0384; Fig. 3c). Further evaluation of forward scatter and CD11b surface marker expression (i.e. characteristics of myelopoiesis and monopoiesis, respectively), showed no significant differences with 1 μM AsIII (Fig. 3d,e).

These results showed that in the K562 cell model, AsIII also inhibits erythropoiesis and megakaryopoiesis, but not myelopoiesis or monopoiesis. The selective inhibition of erythroid and megakaryocytic differentiation and the lack of effects on myelocytic progenitors in two independent cell models (primary mouse bone marrow HPC and K562 cells) suggest that AsIII may be disrupting the function of GATA-1. These findings motivated us to follow-up with mechanistic studies focused on understanding the effects of AsIII on a prominent regulatory ZF transcription factor in erythropoiesis and a critical non-ZF regulator of myelopoiesis, GATA-1 and PU.1, respectively.

### AsIII inhibits GATA-1 activities in vitro

**Figure 4:**
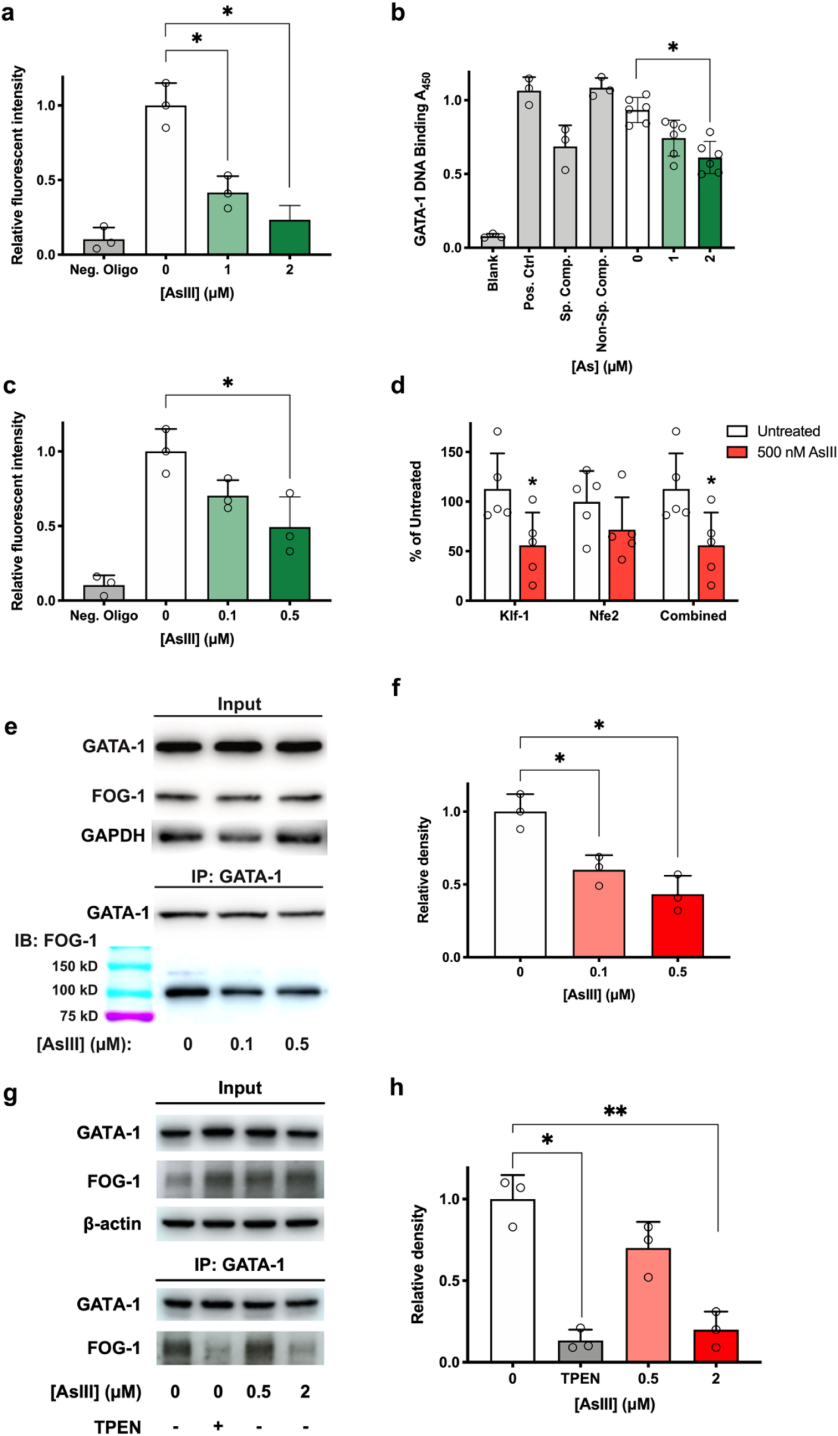
AsIII impairs GATA-1 DNA binding and FOG-1 interaction activities. K562 cells were treated with 0, 1 or 2 µM AsIII for 48 h (**a**,**b**,**g**), and primary mouse bone marrow erythroid cells were treated with 0, 0.1 or 0.5 μM AsIII for 24 h (**c**,**d**,**e**). (**a**) GATA-1 DNA binding measured with an ELISA Protein-DNA Binding Assay Kit in K562 cells. (**b**) GATA-1 DNA binding activity determined by colorimetric Human GATA-1 Transcription Factor Activity Assay in K562 cells. (**c**) GATA-1 DNA binding measured with ELISA Kit in primary bone marrow erythroid cells. (**d**) GATA-1 DNA binding activity analyzed by chromatin immunoprecipitation coupled with qPCR of two GATA-1 regulated genes, *Klf-1* and *Nfe2* (or the combination of both sites). Data are expressed as percentage of untreated control and were derived from the fold enrichment of GATA-1 binding at each site over negative control primers. (**e**) GATA-1 and FOG-1 were co-immunoprecipitated using GATA-1 antibody after AsIII exposure in primary mouse bone marrow erythroid cells. Western blotting of FOG-1 interaction with GATA-1. (**f**) Densitometry of FOG-1 western blotting in primary mouse bone marrow erythroid cells. (**g**) Western-blotting analysis of GATA-1 and FOG-1 binding in K562 cells using co-immunoprecipitation. TPEN is a zinc chelator. (**h**) Densitometry of FOG-1 western blotting in K562 cells. Data are expressed as mean ± SD; *n* = 3 for panels a, c, f, and h; *n* = 6 for panel b; for panel d, *n* = 5–6 (*Klf-1* and *Nfe2*) or *n* = 10–11 (combined). Panels a–c,f,h: *p < 0.05, **p < 0.01 in one-way ANOVA, followed by Tukey’s post hoc test compared to no treatment group. Panel d: *p < 0.05 in two-tailed Student’s *t*-test compared to no treatment group.

Since erythroid and megakaryocyte differentiation depend on the activity of the ZF transcription factor GATA-1^15,59^, we tested the impact of AsIII on GATA-1 DNA and protein binding. GATA-1 DNA binding activity was determined by immunoprecipitating GATA-1 from K562 cells or erythroid differentiated mouse bone marrow HPC and assessing the ability of GATA to bind a GATA consensus sequence using the EpiQuik General Protein-DNA Binding Assay Kit. In 1 and 2 μM AsIII treated K562 cells, GATA-1 DNA binding activity was dramatically decreased by over 50% and 70%, respectively (Fig. 4a). Using the RayBiotech Human GATA-1 Transcription Factor Activity Assay Kit, we observed the same trend of decline of GATA-1 activity (Fig. 4b). The DNA binding activity of GATA-1 was even more sensitive in primary mouse bone marrow erythroid cells with an inhibition of GATA-1 DNA binding found with very low concentrations of 0.1 and 0.5 μM AsIII (30% and 50%, respectively) (Fig. 4c). Further confirmation of the AsIII-induced impairment of GATA-1 DNA binding activity was found using chromatin immunoprecipitation (ChIP) coupled with quantitative real-time PCR (qPCR) in AsIII treated primary mouse bone marrow erythroid cells (Fig. 4d).

In addition to DNA binding activity, GATA-1 interaction with FOG-1 is also critical for its function as a transcriptional regulator^60^. We tested AsIII effects on the interaction between GATA-1 and FOG-1 using co-immunoprecipitation followed by western blotting. A significant inhibition of GATA-1 and FOG-1 interaction was found in primary mouse bone marrow erythroid progenitors starting after 24 h exposure to 0.1 and 0.5 µM AsIII (*p* = 0.0298 and 0.0211, respectively; Fig. 4e,f and Supplementary Fig. S4). Consistently, a suppression of GATA-1 and FOG-1 interaction was also found in K562 cells starting from 0.5 μM AsIII (Fig. 4g). At 2 μM concentration, AsIII reduced the interaction between GATA-1 and FOG-1 by ~ 75% (Fig. 4h). To demonstrate that AsIII inhibition of GATA-1 is through a zinc dependent mechanism, we used the specific zinc chelator, TPEN to demonstrate the necessity of zinc for GATA-1/FOG-1 interaction. We found that treating K562 cells with TPEN markedly decreased GATA-1/FOG-1 interaction (Fig. 4g,h and Supplementary Fig. S5), suggesting that AsIII treatment is functionally equivalent to zinc chelation in terms of removing zinc from GATA-1 protein and inhibiting GATA-1/FOG-1 interaction.

Since AsIII treatment decreased GATA-1 DNA binding and interaction with FOG-1, these findings suggest that AsIII disrupts the function of both the C- and N-terminal ZF domains of GATA. Our results show that AsIII inhibits GATA-1 DNA binding and protein–protein interaction activities, demonstrating GATA-1 as a molecular target of AsIII.

### AsIII interacts with the ZF motif of GATA-1, but not that of PU.1

**Figure 5:**
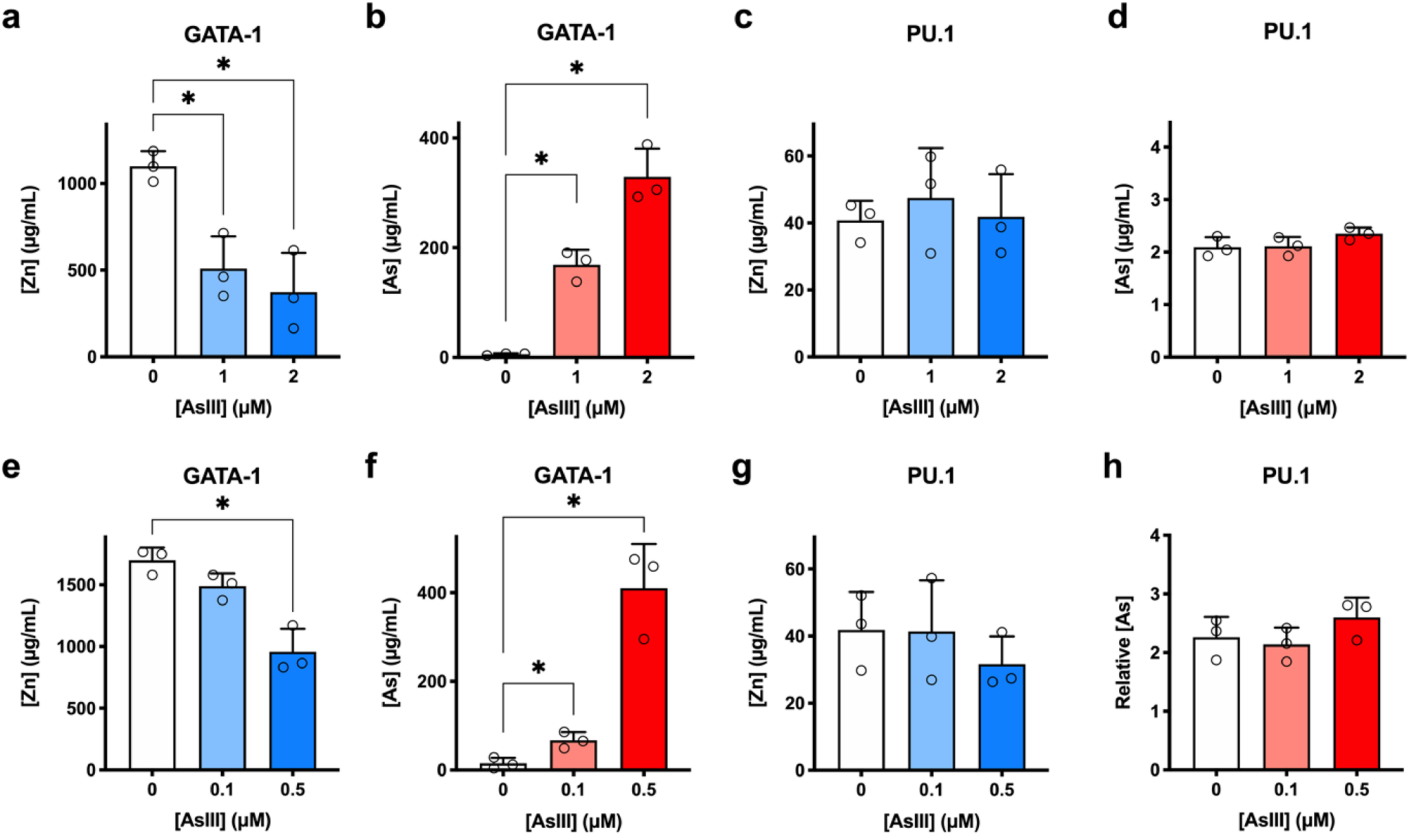
AsIII binds to GATA-1 causing zinc loss but does not bind to PU.1. GATA-1 and PU.1 were immunoprecipitated and zinc/arsenic contents were analyzed by ICP-MS. (**a**) Zinc content in GATA-1 from K562 cells treated with 0, 1 or 2 μM AsIII for 48 h. (**b**) Arsenic bound to GATA-1 protein in AsIII treated K562 cells. (**c**) Zinc and (**d**) arsenic content in PU.1 collected from AsIII treated K562 cells. (**e**) Zinc content in GATA-1 from primary mouse bone marrow erythroid cells after 24 h exposure to 0, 0.1 or 0.5 μM AsIII. (**f**) Arsenic bound to GATA-1 from primary mouse bone marrow erythroid cells. (**g**) Zinc and (**h**) arsenic content in PU.1 from primary mouse bone marrow erythroid cells. Data are expressed as mean ± SD, *n* = 3, *p < 0.05 in one-way ANOVA, followed by Tukey’s post hoc test compared to no treatment group.

We demonstrated that AsIII compromises erythropoiesis and inhibited GATA-1 activity (i.e. DNA binding and FOG-1 interaction), possibly resulting from impairments of the ZF motifs of GATA-1. To demonstrate that the AsIII-induced inhibition of GATA-1 is through ZF disruption, we treated K562 cells and primary mouse bone marrow erythroid progenitor cells with AsIII for 48 and 24 h, respectively. GATA-1 and PU.1 protein was purified from cell lysates using immunoprecipitation and the zinc and arsenic contents were measured by inductively coupled plasma-mass spectrometry (ICP-MS). In K562 cells treated with 1 or 2 μM AsIII, zinc contents were decreased by over 50% (Fig. 5a) and arsenic content was considerably increased (Fig. 5b). GATA-1 from primary mouse bone marrow erythroid progenitor cells showed greater sensitivity to AsIII. Zinc content in GATA-1 was decreased at 0.1 and 0.5 μM AsIII, with 0.5 μM reducing GATA-1 zinc content by approximately 50% (Fig. 5e). Similar to K562 cells, a drastic increase in GATA-1 arsenic content was observed (Fig. 5f). In contrast, AsIII did not modulate zinc content in PU.1, nor bind to PU.1 in either K562 or primary mouse bone marrow erythroid progenitor cells (Fig. 5c,d,g,h), indicating that PU.1 is not a molecular target of AsIII.

Collectively, these results demonstrated that AsIII interacts with GATA-1, reducing zinc content and thus inhibiting DNA and protein binding activities (Fig. 4). More importantly, the interaction of AsIII with GATA-1 was ZF specific, as no changes to PU.1 were found.

### AsIII causes GATA-1 zinc finger dysfunction in vivo

**Figure 6:**
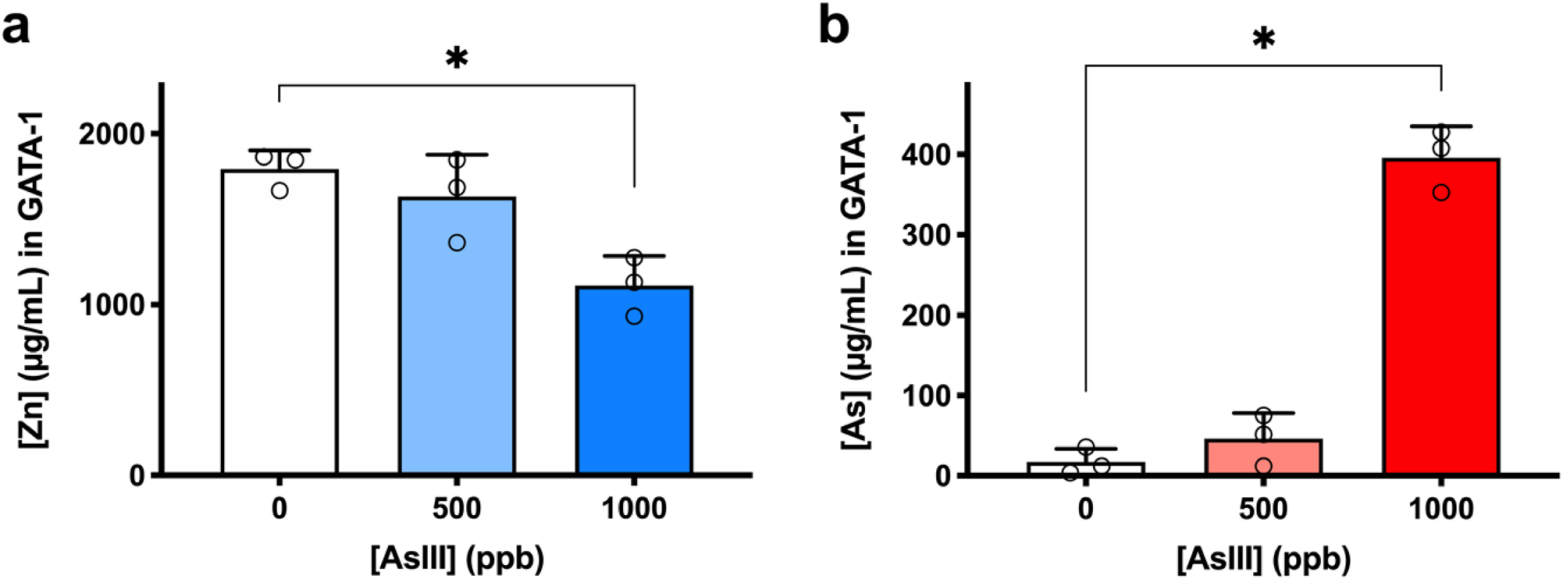
AsIII disrupts GATA-1 zinc finger in vivo. Male C57BL/6J mice were exposed to 500 and 1000 ppb AsIII (6.7 and 13.3 µM) in drinking water for 2 weeks. (**a**) Zinc content and (**b**) arsenic content were measured in GATA-1 immunoprecipitated from bone marrow cells by ICP-MS. Data are expressed as mean ± SD, *n* = 6 mice/group, *p < 0.05 in one-way ANOVA, followed by Tukey’s post hoc test compared to no treatment group.

To further demonstrate that GATA-1 is a sensitive molecular target of AsIII in vivo, we measured arsenic binding and zinc loss using GATA-1 protein collected from bone marrow of C57BL/6J mice exposed to 500 and 1000 ppb AsIII (6.7 and 13.3 µM) via drinking water for 2 weeks. GATA-1 protein was immunoprecipitated from bone marrow cells and ICP-MS was performed to measure zinc and arsenic content in GATA-1. Consistent with findings from our in vitro studies, zinc content in GATA-1 was significantly decreased with 1000 ppb AsIII (*p* = 0.0477; Fig. 6a). We also found a substantial increase of arsenic content in GATA-1 from mice exposed to 1000 ppb AsIII (Fig. 6b). These results corroborated our in vitro findings and provided additional evidence that AsIII interacts with GATA-1 to cause zinc loss and protein dysfunction in vivo.

## Discussion

In this study, we found that AsIII selectively inhibits erythropoiesis, but not myelopoiesis. A prominent factor that contributes to this effect is GATA-1. GATA-1 is a ZF protein with two ZF domains, whereas PU.1 is not a ZF protein. The N-terminal ZF of GATA-1 is responsible primarily for binding with FOG-1 and the C-terminal ZF is responsible for DNA binding^18^. Our previous work in DNA repair inhibition by AsIII demonstrated that ZF proteins such as PARP-1 are sensitive molecular targets of AsIII^42^. We found that AsIII selectively interacts with ZF motifs with ≥ 3 cysteine residues^45^, which are a minority of ZF proteins. Both N-and C-terminal ZFs of GATA-1 contain 4 cysteine residues, making GATA-1 structurally favorable for arsenic binding. In contrast, PU.1 contains only two spatially separated cysteine residues and does not have zinc in its structure. As a result, AsIII does not interact with PU.1.

Our results showed that AsIII exposure not only impaired GATA-1 DNA binding, but also inhibited the interaction of GATA-1 with FOG-1, indicating that AsIII likely binds to both ZFs of GATA-1. Future studies will focus on demonstrating the precise interactions of AsIII with the zinc finger motifs in the GATA-1 protein. Interaction with FOG-1 and DNA binding are both necessary functions of GATA-1 for commitment of HPC to the erythroid lineage and promotion of normal erythropoiesis^18,60^. Previous studies report that a single germ-line mutation on just one ZF of GATA-1 leads to dyserythropoietic anemia^25,26^. Additionally, a mutation in the N-terminal ZF of GATA-1 was found to result in X-linked thrombocytopenia and β-thalassemia^61^. These reports suggest that disruption of either ZF on GATA-1 can cause dysfunction resulting in hematological disorders. Our results suggest that AsIII impairs both ZF motifs on GATA-1, likely explaining why such low concentrations of AsIII cause an inhibition of erythropoiesis.

**Figure 7:**
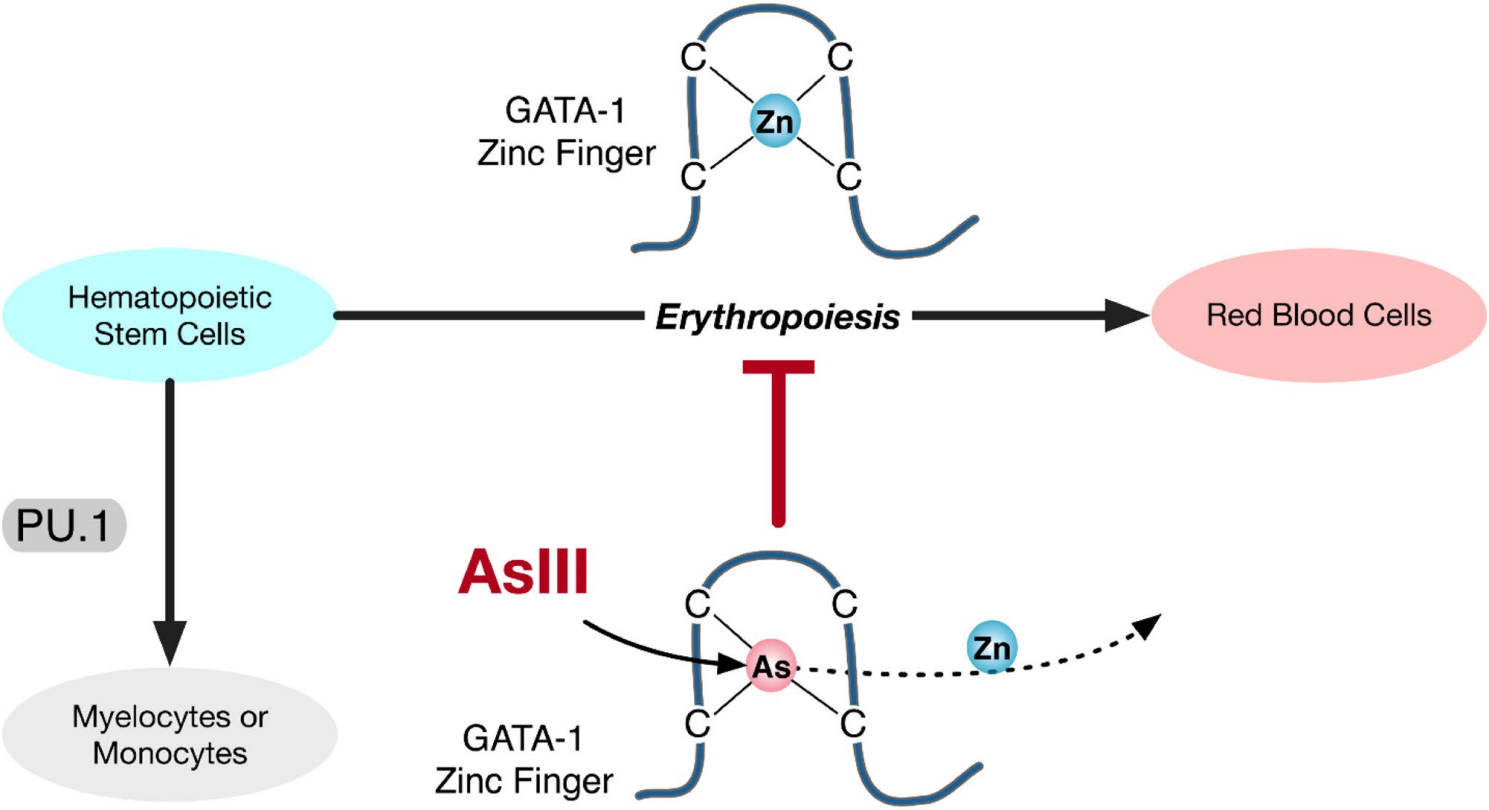
AsIII inhibits erythropoiesis, but not myelopoiesis through interactions with the zinc finger transcription factor, GATA-1. AsIII interacts with the zinc finger motifs (C4) of GATA-1 causing zinc loss and inhibition of DNA and protein binding activities. However, AsIII does not interact with PU.1, a non-zinc finger transcription factor important for myeloid differentiation. The selective effect of AsIII on GATA-1 versus PU.1, results in dyserythropoiesis and produces a shift in the differentiation fate of hematopoietic progenitor cells from erythropoiesis in favor of myelopoiesis.

The interaction of AsIII with GATA-1 not only impaired erythropoiesis, but also produced a shift from erythropoiesis in favor of myelopoiesis (Fig. 7). The lineage commitment of CMPs is regulated by the functional antagonism of GATA-1 and PU.1^10,11,13,62^. Studies have shown that ectopic expression of GATA-1 or PU.1 blocks myeloid or erythroid differentiation, respectively through the functional repression of gene activation^11,62^. The C-terminal ZF of GATA-1 is essential for suppressing the function of PU.1 by direct interaction with the ETS domain of PU.1^10,28^. The shift from erythropoiesis in favor of myelopoiesis represents a ‘hematopoietic imbalance,’ likely resulting from AsIII-induced disruption of the C-terminal ZF of GATA-1. The hypothesis that AsIII disrupts the lineage commitment of HPC resulting from effects on lineage specific transcription factors requires further investigation and will be the topic of our future studies.

In this study we focused on GATA-1 because in addition to being essential for normal erythropoiesis, the peak expression and activity levels of GATA-1 occur during the stages of erythroid differentiation (i.e. BFU-E, CFU-E, proerythroblast)^15,17,18^ which we have found in this and previous studies to be most impacted by AsIII exposure^41^. However, there are other zinc finger proteins which have important roles during hematopoiesis that may also be impacted by arsenic exposure^63^. In particular, GATA-2 is a zinc finger transcription factor, which is similar to GATA-1, contains two C4 zinc finger motifs^64,65^. GATA-2 is highly expressed in HSC and has essential regulatory functions during hematopoiesis^65–70^, in part through its activity in promoting the erythroid lineage commitment of HPC through the activation of GATA-1 expression^71,72^. The effects on GATA-1 observed in this study appear to be mediated through interactions of AsIII with the ZF motifs of GATA-1, as no effects on GATA-1 expression levels were found. It is plausible, however, that indirect effects mediated through other zinc finger proteins (e.g. GATA-2) may also contribute to the disruption of erythropoiesis, and as such will be a focus of future investigations.

In this work, the concentrations of AsIII that produced an inhibition of erythropoiesis started at 20 ppb (~ 0.3 µM), which is lower than 12% of the AsIII concentrations measured in water supplies in north central and western regions of the United States^33,34^. Some unregulated water sources in rural areas around the world contain much higher AsIII levels^33,34^. Many regions with high endemic AsIII are also reported to be disproportionately affected by anemia, even after statistical adjustment for common risk factors^35–37,39^. These studies emphasize the necessity for gaining a deeper mechanistic understanding of arsenic-caused anemias.

Findings from the present study showed that GATA-1 is a sensitive target of AsIII. GATA-1 ZF disruption was observed with environmentally relevant concentrations of AsIII in vitro and in vivo. We identified a molecular mechanism by which AsIII causes GATA-1 dysfunction producing effects functionally similar to germ-line mutations of GATA-1. Such mutations of GATA-1 ZF result in severe dyserythropoietic anemia^25,26^. Although inherited dyserythropoietic anemia is rare, millions of people worldwide are chronically exposed to AsIII through drinking water. This presents a very important environmental health issue that requires further attention from the scientific community.

Our work demonstrates for the first time that AsIII, a widespread environmental toxicant, inhibits erythropoiesis through disruption of the ZF transcription factor, GATA-1. AsIII disrupts the function of GATA-1 likely through interactions with its ZF motifs, resulting in dyserythropoiesis and an imbalance of HPC differentiation. These findings present a novel mechanism by which AsIII may cause anemia and provide critical insights into potential prevention and intervention for arsenic and other environmental exposure related anemias.

## Methods

### Chemicals and in vitro cell dosing

Sodium meta-AsIII (> 95% purity), hemin, and phorbol 12-myristate 13-acetate (PMA) were purchased from Sigma-Aldrich. AsIII stock solutions for in vitro experiments were prepared fresh at the time of use in cell culture medium. Depending on the experiment and cell type (i.e. K562 cells or primary mouse bone marrow HPC), AsIII exposure concentrations were 0.1 (7.5 ppb), 0.5 (37.5 ppb), 1 (75.0 ppb), or 2 μM (150.0 ppb) as indicated in figure legends.

### Cell culture and chemical-induced differentiation of K562 cells

The human leukemic cell line, K562 was purchased from ATCC. K562 cells were maintained in Dulbecco’s Modified Eagle Medium with 10% FBS according to instructions from ATCC. Erythroid, megakaryocytic, myelocytic, and monocytic differentiation of K562 cells was induced by 24 h treatment with 5 μM hemin or 5 ng/mL PMA as described previously^54–57^. K562 cell differentiation was determined by flow cytometry using the following monoclonal antibodies (all from ThermoFisher Scientific): erythropoietic (CD71-APC, CD235a-PE), megakaryocytic (CD41-APC), myelocytic (CD11b-FITC), and monocytic (CD11b and forward scatter)^54–57^. See below for a detailed description of flow cytometry staining procedure.

### Mice and in vivo drinking water exposures

Experiments involving mice were performed in accordance with protocols approved by the Institutional Animal Use and Care Committee at the University of New Mexico Health Sciences Center. All experiments were performed in accordance with relevant guidelines and regulations. Male C57BL/6J mice were purchased from Jackson Laboratory and acclimated for one week prior to in vivo and in vitro experiments. All mice used for in vitro and in vivo studies were approximately 12–13 weeks of age. Male mice were used in this study based on our reported observation of a decreased red blood cell counts in a group of men from rural Bangladesh who were exposed to AsIII in their drinking water^38^, to avoid any potential complication due to gender difference. Mice were maintained on a 12:12 reverse light:dark cycle and fed ad libitum 2920X Teklad rodent diet (Envigo). Mice were exposed via their drinking water to 0 (no treatment), 20 (0.3 μM), 100 (1.3 μM), and 500 ppb (6.7 μM) or to 0, 500 ppb, and 1000 ppb (13.3 μM) AsIII for 30 days (*n* = 5 mice/group) or 2 weeks (*n* = 6 mice/group), respectively.

### Primary mouse bone marrow cell isolation

Bone marrow cells were isolated from femur bones as described by Ezeh et al.^73^. Femurs from each mouse were flushed using a 1 mL syringe and 25-G needle. The cell suspension was transferred to a 15 mL centrifuge tube, centrifuged at 200 *g* for 10 min, and resuspended in 20 mL of Isocove’s Modified Dulbeccos Medium ((IMDM) with 2 or 10% FBS, 20 mM L-glutamine, and 100 mg/ml streptomycin and 100 units/mL penicillin). Cell viabilities and concentrations were determined using acridine orange/propidium iodide (AO/PI) staining with a Nexcelom Cellometer Auto 2000 (Nexcelom Bioscience).

### CFU-E assay

Mouse CFU-E assays was performed according to manufacturer’s instructions (STEMCELL Technologies Technical Manual for Mouse Colony-Forming Unit Assays using MethoCult version 3.4.0). Bone marrow cells (4 × 10^5^) in IMDM with 2% FBS were transferred into 4 mL MethoCult M3334 methylcellulose-based medium containing EPO (STEMCELL Technologies). Samples were vortexed thoroughly and transferred in triplicate (1 × 10^5^ cells) to treated 35-mm culture dishes (STEMCELL Technologies). CFU-E colonies were enumerated after 72 h using an inverted microscope. CFU-E colony counts are reported as number of colonies/10^6^ bone marrow cells.

### Hematopoietic progenitor cell isolation and in vitro erythropoiesis model

HPC were isolated from bone marrow using the EasySep Mouse HPC Isolation Kit (STEMCELL Technologies) according to manufacturer’s instructions. Bone marrow cells were concentrated to 1 × 10^8 ^cells/mL in Easy Sep Buffer (DPBS without calcium and magnesium (DPBS^−^) containing 2% FBS and 1 mM EDTA) and stained with a cocktail of biotinylated lineage-specific antibodies (CD5, CD11b, CD19, CD45R/B220, Ly6G/C(Gr-1), TER119) for 15 min at 4 °C. Streptavidin-coated magnetic particles were added to each sample and incubated for 10 min. Samples were brought up to 2.5 mL in EasySep buffer and placed into an EasySep magnet (STEMCELL Technologies) for isolation. Supernatant containing HPC was collected and utilized to develop an in vitro model of erythropoiesis as described by Shuga et al.^51^. HPC were cultured in SF StemSpan hematopoietic progenitor expansion media (STEMCELL Technologies) containing AsIII and supplemented with 100 ng/mL murine stem cell factor (SCF) and 5 IU/mL (31.25 ng/mL) human recombinant EPO (Peprotech) to stimulate erythroid lineage commitment and differentiation. Erythroid progenitor cells were continuously exposed to AsIII throughout differentiation.

### Flow cytometry

Erythroid and myeloid progenitors were evaluated based on surface marker phenotype as defined by Pronk et al.^52^ and Grover et al.^53^: CMP (Lin^−^, cKit^+^, SCA-1^−^, CD16/32^−^, CD34^+^) Pre-Megakaryocytic-Erythroid Progenitor (PreMegE; Lin^−^, cKit^+^, SCA-1^−^, CD16/32^−^, CD150^+^, CD105^−,low^); MEP (Lin^−^, cKit^+^, SCA-1^−^, CD16/32^−^, CD34^−^), BFU-E (Lin^−^, cKit^+^, SCA-1^−^, CD16/32^−^, CD150^+^, CD105^+^); CFU-E (Lin^−^, cKit^+^, SCA-1^−^, CD16/32^−^, CD150^−^, CD105^+^); Pre-GM; Lin^−^, cKit^+^, SCA-1^−^, CD16/32^−^, CD150^−^, CD105^−^); Granulocyte Macrophage Progenitor (GMP; Lin^−^, cKit^+^, SCA-1^−^, CD16/32^+^, CD150^−^). Erythroblast differentiation was evaluated using CD71 and Ter119 surface marker expression and cell size characteristics (early erythroblasts, CD71^low/high^, Ter119^−,low^; basophilic erythroblasts (EryA), CD71^high^Ter119^high^FSC^high^; late basophilic and polychromatic erythroblasts (EryB), CD71^high^Ter119^high^FSC^low^; and orthochromatic erythroblasts (EryC), CD71^low^Ter119^high^FSC^low^) as previously described by Socolovsky et al.^49^ and Koulnis et al^50^.

For surface marker analysis, 0.5–1 × 10^6^ cells were stained with 0.5 µg of monoclonal antibodies (all from BD Biosciences): cKit-APC-R700, CD34-PE-Cy7, SCA-1-BV605, CD16/32-BV510, CD150-BV421, CD105-BB515, CD71-PE and Ter119-FITC in 100 µL BD Horizon Brilliant Stain Buffer at room temperature in the dark for 30 min. Samples were washed twice and resuspended in 0.5 mL DPBS^−^ with 2% FBS and 0.09% sodium azide prior to analysis using a BD Accuri C6 or BD LSRFortessa flow cytometer. Gates were set with the aid of fluorescence-minus-one controls. Flow cytometry data are reported as percentages or absolute cell numbers. Absolute cell numbers were generated by multiplying population subset percentages by total viable cell counts (for cell counts, see Supplementary Tables S1, S2).

### Immunoprecipitation and co-immunoprecipitation

GATA-1 or PU.1 protein was isolated by immunoprecipitation and GATA-1/FOG-1 complex was co-immunoprecipitated as previously described^45^. Briefly, GATA-1, FOG-1, or PU.1 were purified using GATA-1 (D52H6) XP rabbit monoclonal antibody, PU.1 (9G7) rabbit monoclonal antibody (Cell Signaling Technologies), or FOG-1 rabbit polyclonal antibody (ab86281; Abcam), respectively using Dynabeads Protein-A Immunoprecipitation Kit (ThermoFisher Scientific). Immunoprecipitated GATA-1, FOG-1, and PU.1 were eluted from the beads and utilized for downstream experiments. GATA-1 and FOG-1 interaction was determined in co-immunoprecipitated GATA-1/FOG-1 complex by western blotting. Densitometry summaries were obtained from 3 independent blots after normalization to the density level of the whole image. To prepare samples for ICP-MS, a non-denaturing method was used to elute GATA-1 or PU.1 from beads, and the solution adjusted to pH > 7 using the neutralizing buffer provided in the immunoprecipitation kit.

### GATA-1 DNA binding activity

GATA-1 DNA binding activity was determined using the fluorometric EpiQuik General Protein-DNA Binding Assay Kit (EpiGentek) according to the manufacturer’s instructions. GATA-1 DNA binding activity was determined with immunoprecipitated GATA-1 by assessing the ability of GATA to bind a GATA consensus double-stranded DNA probe sequence^74^ (GCCCCCGCTGATTCCCTTATCTATGCCTTCCCAGC) or negative control double-stranded DNA probe sequence (ACAGGGATGGGGGAGGGAATGGGGTGAGGCCTGTC) using the EpiQuik General Protein-DNA Binding Assay Kit (Epigentek). All procedures strictly followed the instruction except 1:500 dilution of GATA-1 antibody was used. Nuclear extracts were prepared using the EpiQuik Nuclear Extraction Kit according to manufacturer’s instructions (Epigentek). Fluorescence intensity was measured with a plate reader at Ex. 495 nm and Em. 520 nm. Human GATA-1 Transcription Factor Activity Assay (RayBiotech) was also used. Following erythroid differentiation, nuclear proteins were extracted from each treatment group (1.2 × 10^7^ cells/group) using the Nuclear Extraction Kit according to instructions from the manufacturer (RayBiotech). Isolated nuclear proteins were then loaded into the wells of 96-well plate coated with double stranded oligonucleotides containing GATA-1 binding sequences. The Raybiotech Kit provides specific and non-specific competitor DNA oligos. Positive control (cell extract) were used with each of the competitor DNA oligos as control in the DNA binding assay. GATA-1 binding activity was determined using a primary antibody against GATA-1. Following primary antibody incubation, HRP-conjugated secondary antibody was added, and colorimetric signal was measured with a spectrophotometric plate reader at 450 nm.

### ChIP assay and qPCR

GATA-1 DNA binding activity was determined using the ChIP-IT High Sensitivity and ChIP-IT Control qPCR Kits (Active Motif) according to manufacturer's instruction with some modifications. Briefly, ~ 2–3 × 10^6^ mouse bone marrow erythroid progenitor cells were used for the ChIP assay. Crosslinking and cell lysis were performed in accordance with the ChIP-IT High Sensitivity Kit manual. Chromatin was sheared on ice using a micro ultrasonic cell disrupter (Kontes) to approximately 200–1000 bp. Chromatin fragment size was confirmed by analyzing size distribution in a 1.5% agarose gel. All immunoprecipitation, decrosslinking, and DNA purification steps were performed as described by the manufacturer. For immunoprecipitation, 7 µg of sheared chromatin was combined with ChIP grade GATA-1 antibody (4 µg; ab11852; Abcam), normal mouse IgG (2 µg; Active Motif) or RNA Polymerase II + bridging antibody (2 µg each; Active Motif). All assays exceeded the quality control criteria established by Active Motif for the ChIP-IT High Sensitivity Kit.

Following ChIP procedure, purified DNA was utilized to analyze the enrichment of GATA-1 binding by qPCR using the ChIP-IT qPCR Analysis Kit (Active Motif). Primers were designed to regions of the *Klf-1* and *Nfe2* genes as described by Suzuki et al.^72^ The sequences used were cross referenced to available ChIP-sequencing data from the University of California Santa Cruz Genomics Institute Encyclopedia of DNA Elements to verify that they were valid genomic regions for evaluation of GATA-1 DNA binding activity (Supplementary Figs. S2, S3; https://genome.ucsc.edu/ENCODE/index.html).

Primer sequences are listed in Supplementary Table S3. Primers efficiencies were tested and found to be ~ 100% for both *Klf-1* and *Nfe2* primer sets. In addition, universal negative control primers (*Gapdh2)* provided in the ChIP-IT qPCR Analysis Kit (Active Motif) were utilized for data analysis. Likewise, all other qPCR conditions were optimized. qPCR was performed with SsoAdvanced Universal SYBR Green Supermix (BioRad) according to manufacturer’s instructions using a using a BioRad CFX384 Touch Real-Time PCR Detection System (BioRad). In an effort to minimize inter-sample variability, results from two independent experiments were expressed as fold enrichment over negative control primers, converted to percentage of untreated control, and merged for subsequent data analysis.

### Inductively coupled plasma-mass spectrometry

Immunoprecipitated GATA-1 or PU.1 protein was collected, and protein content determined using the Pierce BCA Protein Assay Kit (ThermoFisher Scientific). Protein samples were diluted in trace metal grade nitric acid and zinc and arsenic contents in GATA-1 or PU.1 were measured by ICP-MS. Blank samples, internal standards, and standard curves were included with experimental samples as quality control for preparation and analyses. Data were calculated from the standard curve values generated during each experiment and values generated from negative quality control samples were subtracted as background prior to data analysis. Results were normalized to immunoprecipitated protein content.

### Statistics

Flow cytometry data was processed using FlowJo version 10 (FlowJo LLC). Data were analyzed with GraphPad Prism 7. Differences between no treatment and treatment groups were determined using one-way ANOVA, followed by Tukey's post hoc test or two-tailed Student’s *t*-test at a significance level of *p* < 0.05 or lower, as indicated in figure legends. All results from K562 cells are biological replicates and are representative of one of three independent experiments. Results from primary bone marrow HPC include at least three technical replicates and are representative of one of three independent experiments.

## Supplementary information

Supplementary Information

## Data Availability

The datasets generated/analyzed during the current study are available from the corresponding author (Ke Jian Liu: kliu@salud.unm.edu) on reasonable request.
