# Supplementary material for: Inhibition of red blood cell development by arsenic-induced disruption of GATA-1
Source: Sci Rep. 2020 Nov 4;10:19055. doi: 10.1038/s41598-020-76118-x (PMC7643154; doi:10.1038/s41598-020-76118-x)
Supplement: Supplementary file 1 — Supplementary Information [file 41598_2020_76118_MOESM1_ESM.docx]

**Supplementary Materials**

**Inhibition of red blood cell development by arsenic-induced disruption of GATA-1**

Xixi Zhou, Sebastian Medina, Alicia M. Bolt, Haikun Zhang, Guanghua Wan, Huan Xu, Fredine T. Lauer, Shu Chun Wang, Scott W. Burchiel, and Ke Jian Liu*

**Table S1.** Total viable bone marrow cell recoveries (per femur set)^1,2,3^.

| **Time Point** | **Control, ×10^6^** | **0.1 µM AsIII, ×10^6^** | **0.5 µM AsIII, ×10^6^** |
| --- | --- | --- | --- |
| 24 h | 0.40 ± 0.04 | 0.37 ± 0.05 | 0.38 ± 0.02 |
| 48 h | 1.26 ± 0.06 | 1.18 ± 0.05* | 0.88 ± 0.04* |
| 72 h | 2.03 ± 0.19 | 1.69 ± 0.10* | 1.25 ± 0.10* |

^1^Mice were exposed to 0, 20, 100, and 500 ppb AsIII via their drinking water for 30 days.

^2^Viable cells were measured using acridine orange/propidium iodide staining and a Nexcelom Cellometer Auto 2000.

^3^Data are expressed as mean ± SD, *n* = 5.

**Table S2.** Total viable primary mouse bone marrow hematopoietic progenitor cells (stimulated with EPO and SCF) measured following 24, 48, and 72 h AsIII exposure^1,2,3^.

| **AsIII Dose Group** | **Total Viable Cells, ×10^6^** |
| --- | --- |
| Control (0 ppb) | 44.91 ± 10.45 |
| 20 ppb | 38.30 ± 13.40 |
| 100 ppb | 36.24 ± 2.68 |
| 500 ppb | 30.47 ± 1.93 |

^1^Cells were plated at approximately 3×10^5^ cells (0 h timepoint).

^2^Viable cells were measured using acridine orange/propidium iodide staining and a Nexcelom Cellometer Auto 2000.

^3^Data are expressed as mean ± SD, *n* = 6, **p*<0.05 in one-way ANOVA, Tukey’s multiple-comparison tests compared to untreated control group.

**Table S3.** ChIP-qPCR primer sequences^1^.

| **Region** | **Sequence** |
| --- | --- |
| *Klf-1* | FWD: 5’–CTGATAGCGGCCTGAAACAT – 3’  REV: 5’– AAGGTCTTTGGGACCCTTTG – 3’ |
| *Nfe2* | FWD: 5’ – CCCTCTCCTCCCATCCTTAC – 3’  REV: 5’ – GGGACATGACCAAATGACCT – 3’ |

^1^Supplementary Figure S2 and S3 depicts the genomic region the primers were designed to amplify.

**Supplementary Figures**

**Supplementary Fig. S1**

**
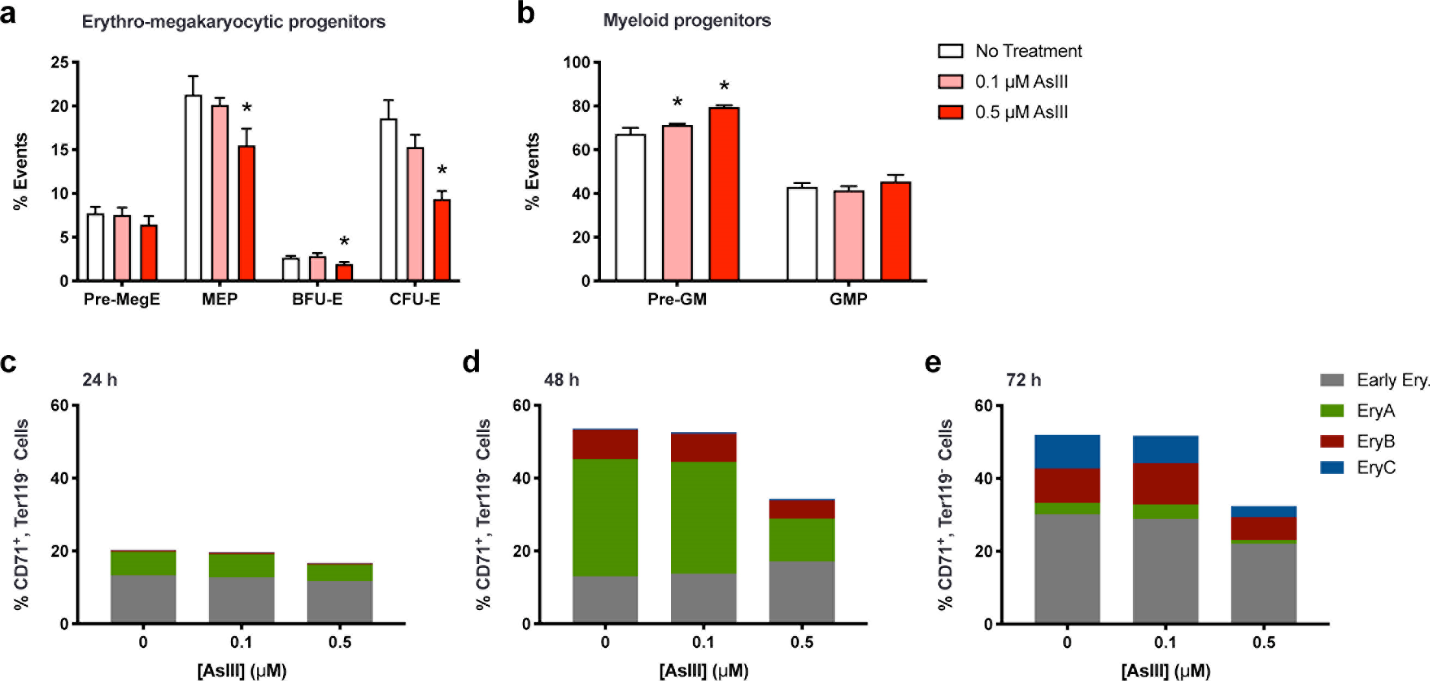
**

**Supplementary Fig. S1.** Analysis of erythro-megakaryocytic and myeloid progenitor subsets following 24 h exposure of primary mouse bone marrow hematopoietic progenitor cells (stimulated with EPO and SCF to promote erythroid differentiation) to 0.1 and 0.5 µM AsIII. (**a**) Percentages of surface marker defined erythro-megakaryocytic progenitors: CMP (Lin^-^, cKit^+^, SCA-1^-^, CD16/32^-^, CD34^+^); PreMegE (Lin^-^, cKit^+^, SCA-1^-^, CD16/32^-^, CD150^+^, CD105^-, low^); MEP (Lin^-^, cKit^+^, SCA-1^-^, CD16/32^-^, CD34^-^), BFU-E (Lin^-^, cKit^+^, SCA-1^-^, CD16/32^-^, CD150^+^, CD105^+^); CFU-E (Lin^-^, cKit^+^, SCA-1^-^, CD16/32^-^, CD150^-^, CD105^+^) and (**b**) myeloid progenitors: Pre-GM (Lin^-^, cKit^+^, SCA-1^-^, CD16/32^-^, CD150^-^, CD105^-^); GMP (Lin^-^, cKit^+^, SCA-1^-^, CD16/32^+^, CD150^-^) after 24 h exposure to 0, 0.1, and 0.5 μM AsIII. (**c-e**) Percentage of erythroblast subsets (early erythroblasts, CD71^low/high^, Ter119^-,low^; basophilic (EryA), CD71^high^Ter119^high^FSC^high^; late basophilic and polychromatic (EryB), CD71^high^Ter119^high^FSC^low^; orthochromatic (EryC), CD71^low^Ter119^high^FSC^low^) after 24, 48, and 72 h exposure to 0, 0.1, and 0.5 μM AsIII. Data are expressed as mean ± SD, *n* =3, **p*<0.05 in one-way ANOVA, Tukey’s post hoc test compared to no treatment control group.

**Supplementary Fig. S2**


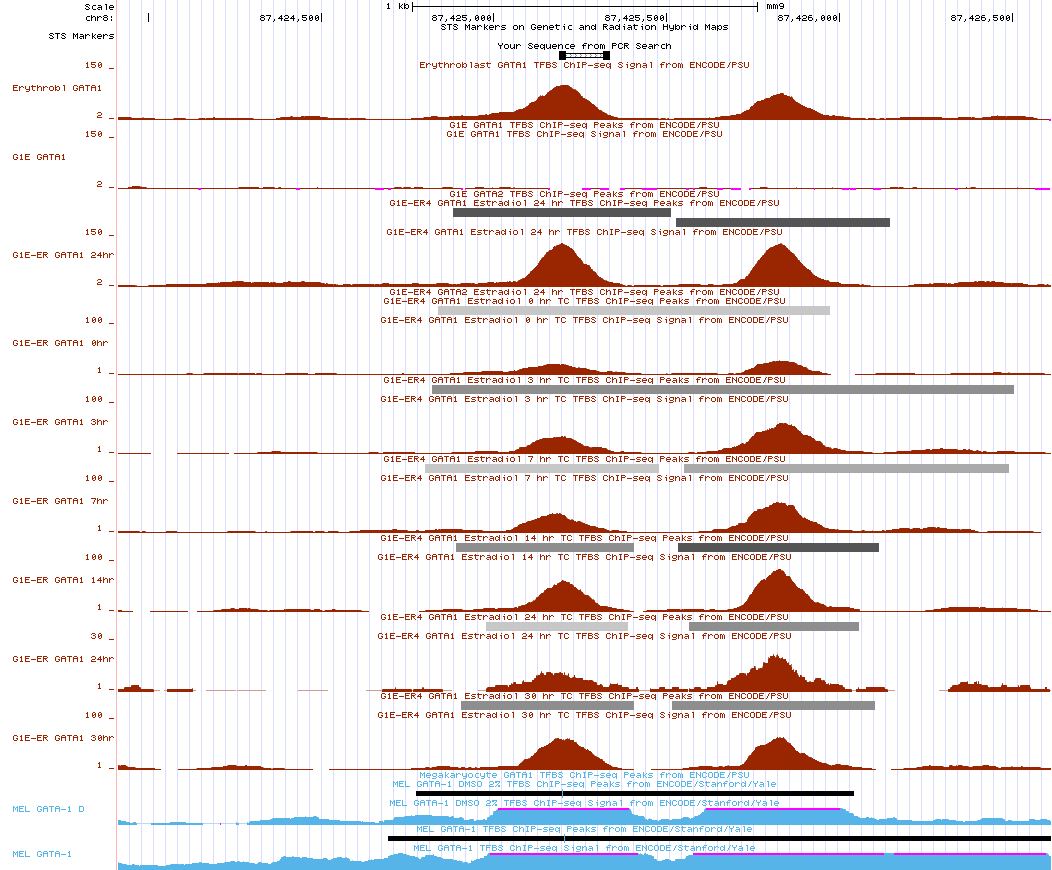


**Supplementary Fig. S2.** Image showing the enrichment of GATA-1 binding at the *Klf-1* sequence used as our target for ChIP-qPCR experiments (indicated with the red box). The y-axis represents available ChIP-seq data from various erythroid progenitor cell types deposited in the University of California Santa Cruz (UCSC) Encyclopedia of DNA Elements Genome Browser on Mouse (<https://genome.ucsc.edu/ENCODE/index.html>). Image was generated using the *in-silico* PCR tool on the UCSC Encyclopedia of DNA Elements webpage with the primer sequences provided in Supplementary Table S3.

**Supplementary Fig. S3**


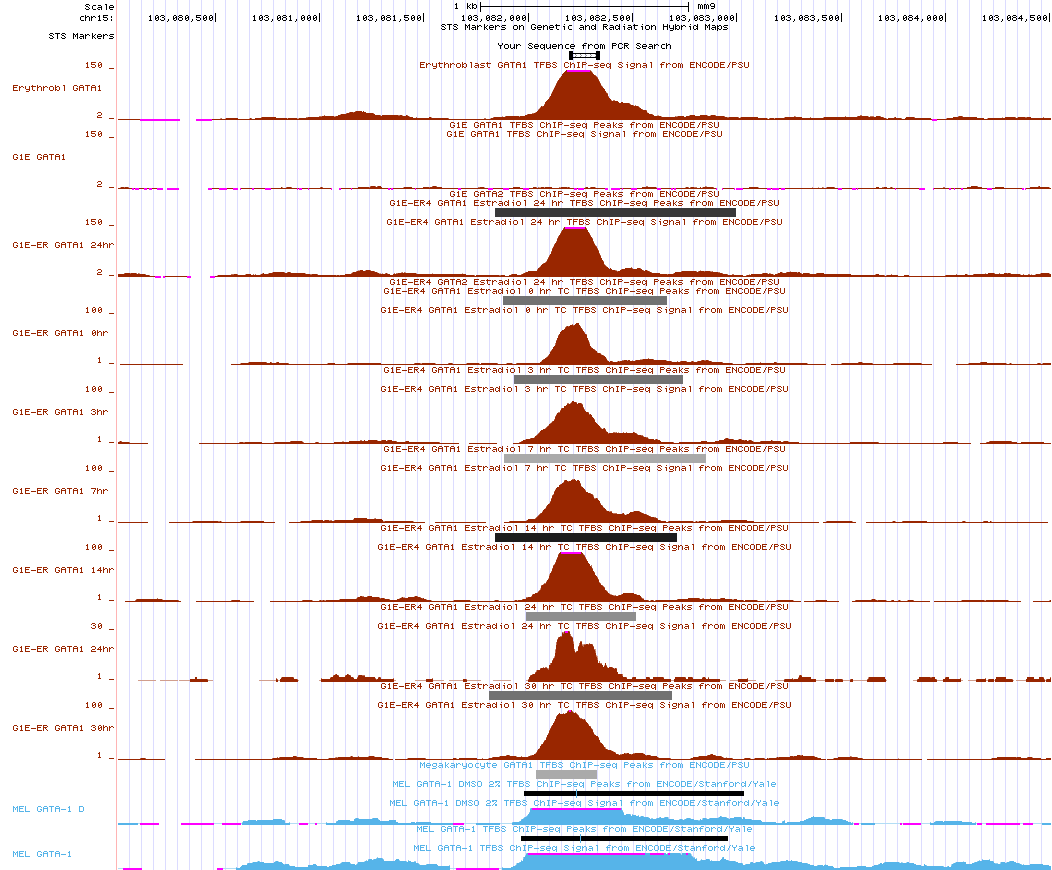


**Supplementary Fig. S3.** Image showing the enrichment of GATA-1 binding at the *Nfe2* sequence used as our target for ChIP-qPCR experiments (indicated with the red box). The y-axis represents available ChIP-seq data from various erythroid progenitor cell types deposited in the University of California Santa Cruz (UCSC) Encyclopedia of DNA Elements Genome Browser on Mouse (<https://genome.ucsc.edu/ENCODE/index.html>). Image was generated using the *in-silico* PCR tool on the UCSC Encyclopedia of DNA Elements webpage with the primer sequences provided in Supplementary Table S3.

**Supplementary Fig. S4**

| **Input:** |  |
| --- | --- |
| **GATA-1** | **FOG-1** |
| **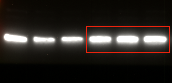** | **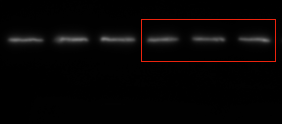** |
|  |  |
| **GAPDH** |  |
| **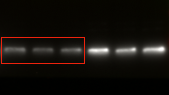** |  |
|  |  |
| **IP:** |  |
| **GATA-1** | **FOG-1** |
| 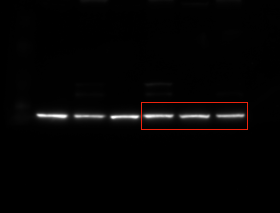 | **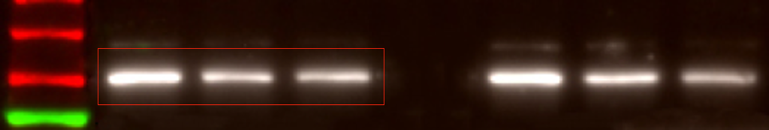** |

**Supplementary Fig. S4.** Full-length blots corresponding to Fig. 4e. Lanes from left to right for all blot images: untreated, 0.1 µM AsIII, or 0.5 µM AsIII.

**Supplementary Fig. S5.**

| **Input:** |  |
| --- | --- |
| **GATA-1** | **FOG-1** |
| 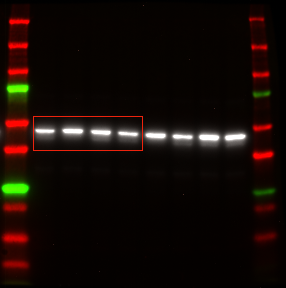 | 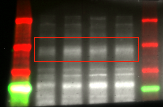 |
|  |  |
| **β-actin** |  |
| 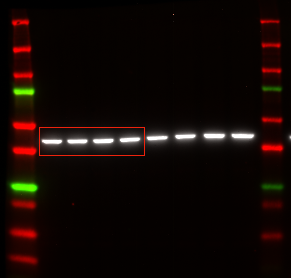 |  |
|  |  |
| **IP:** |  |
| **GATA-1** | **FOG-1** |
| **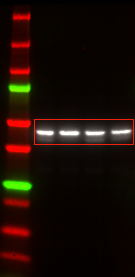** | **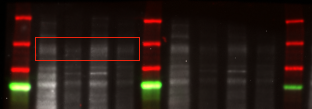** |

**Supplementary Fig. S5.** Full-length blots corresponding to Fig. 4g. Lanes from left to right for all blot images: untreated without the zinc chelator, TPEN, untreated with TPEN, 0.5 µM AsIII, or 2 µM AsIII.
